# Scale-Dependent hydroxyl radical generation and energy efficiency in vortex diode hydrodynamic cavitation: machine learning insights toward industrial-scale applications in water treatment^☆^

**DOI:** 10.1016/j.ultsonch.2026.107932

**Published:** 2026-06-22

**Authors:** Xinzhu Pang, Varaha P. Sarvothaman, Shekhar R. Kulkarni, William L. Roberts, Vivek V. Ranade

**Affiliations:** a School of Forensic Medicine, China Medical University, No 77 Puhe Road, Shenyang North New Area, Shenyang 110122, Liaoning, PR China; b Clean Energy Research Platform (CERP), Physical Sciences and Engineering Division, King Abdullah University of Science and Technology (KAUST), Thuwal 23955-6900, Saudi Arabia; c Multiphase Reactors and Intensification Group, Bernal Institute, University of Limerick, Limerick V94T9PX, Ireland

**Keywords:** Hydroxyl radicals, XGB, ANN, Scale-up, SHAP analysis

## Abstract

Hydrodynamic cavitation (HC) reactors are increasingly applied in the remediation of organic pollutants in water, leveraging intense shear and hydroxyl radical (OH•) generation to accelerate degradation processes. However, scaling up HC devices remains a challenge in environmental engineering due to poorly understood effects at different scales of operation. Vortex-based HC (VDs) provide superior cavitation efficiency compared to traditional orifice and venturi devices and therefore this study examines the scale-dependent generation of OH• in VDs. Coumarin dosimetry was employed as the quantification method for OH• generation. Available published experimental datasets were analysed: (i) varying inlet pressures from 100 to 400 kPa for throat diameters of 6 and 12 mm, and (ii) throat diameters ranging from 6 to 38 mm (nominal capacities of 5 to 200 L/min) at a fixed pressure drop of 280 kPa. After normalization of the available data, seven machine learning (ML) models were trained to establish relationships between operating conditions and OH• generation performance. eXtreme Gradient Boosting (XGB) and artificial neural networks (ANN) outperformed the others, with higher R^2^ and lower RMSE. After using SHAP interpretation, these two models were used to elucidate scale effects on both radical yield and energy efficiency, resulting in actionable design guidelines for VDs at different scales. By combining experimental dosimetry with predictive ML, this work advances the fundamental understanding and practical implementation of cavitation-based advanced oxidation technologies, particularly for efficient and energy-optimized treatment of organic pollutants in wastewater.

## Graphical abstract

**Figure:**
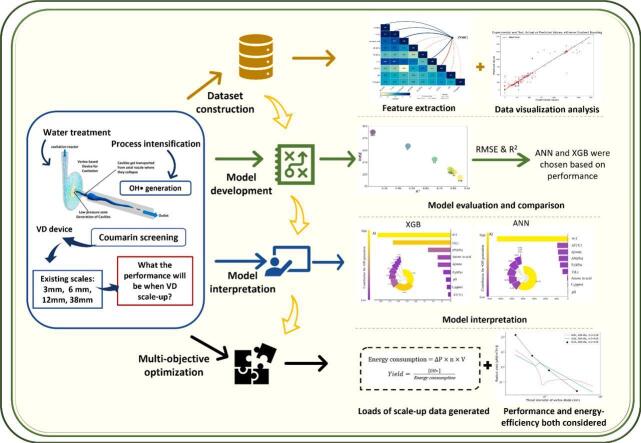

## Notations

**Acronym**
$V_{\mathrm{operation}}$: Volume of operation with the vortex-based HC reactor (L)
$n^{*}$: Characteristic number of passes normalized to a reference operation (−)
$\overset{¯}{y}$: Average value of the target feature, concentration of 7OHC (µM)
$\hat{y}$: Predicted value of the target feature, concentration of 7OHC (µM)
7OHC: Concentration of 7-hydroxycoumarin (µM)
AI: Artificial intelligence
ANN: Artificial Neural Networks
DT: Decision Tree
d_t_: Characteristic throat diameter (mm)
GB: Gradient Boosting
HC: Hydrodynamic cavitation
KNN: K-Nearest Neighbours
LPM: Liter per minutes
LR: Linear Regression
ML: Machine learning
n (−): Characteristic number of passes (−)
P_1_: Inlet pressure to vortex-diode reactor (kPa)
P_2_: Downstream pressure in the vortex-diode reactor (kPa)
RF: Random Forest
VD: Vortex-based HC devices
XGB: eXtreme Gradient Boosting
ΔP: Pressure drop across the vortex-diode based HC reactor (kPa)
M: Total number of data points
Q: Flowrate of vortex-diode based HC reactor (m^3^/s)
m: Data point for an instance
y: Actual value of the target feature, concentration of 7OHC (µM)

## 1 Introduction

Hydrodynamic cavitation (HC) has emerged as a promising, additive-free technology for environmental remediation, particularly for degrading recalcitrant organic pollutants in wastewater. By inducing controlled bubble collapse via pressure gradients in flowing liquids, HC creates extreme localized conditions – temperatures 2000–5000 K, pressures 100–1000 atm, and intense shear forces – that generate highly reactive hydroxyl radicals [1], [2], [3]. These radicals non-selectively oxidize a wide range of contaminants, including dyes [4], [5], [6], pharmaceuticals [7], [8], [9], aromatic contaminants [10], [11], [12] and microplastics [13], often achieving mineralization or conversion to less harmful by-products. Unlike conventional advanced oxidation processes (AOPs) such as UV/H_2_O_2_ or ozonation, HC requires no external chemical, operates at ambient temperature, and integrates easily into existing treatment trains [14]. Its capacity for processing large volumes of wastewater positions it as a promising candidate for industrial-scale applications [1]. However, systematic device selection and scale-up remain challenging because the 'active' cavitating region – where vaporous cavities collapse must be precisely characterized to optimize performance. Orifice, venturi and vortex-diode are the commonly investigated HC reactor types. While numerous studies explore applications of HC, very few studies comparing performance of geometrically similar devices on different scales are available [15], [16], [17], leaving industrial-scale implementation poorly guided. Influence of scale on geometrically similar orifice and venturi at different scales is rarely reported. Some studies using micro-orifice and micro-venturi systems are available [18], [19]. While these studies can provide mechanistic understanding under controlled conditions, these micro-devices operate at very low flow rates and are not relevant for scale-up to industrially relevant systems, which is the focus of this work.

**Fig. 1:**
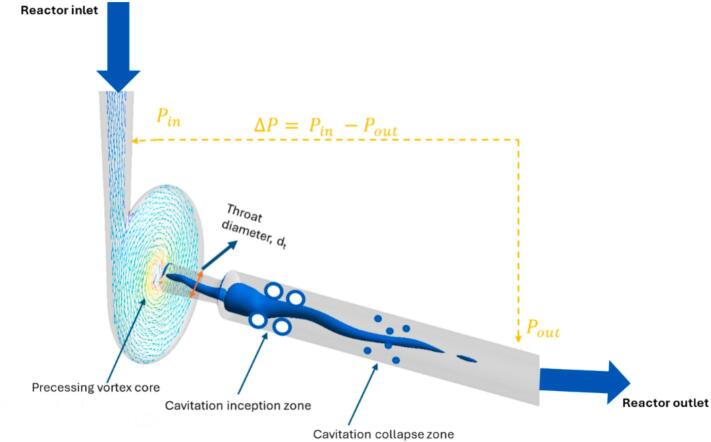
Schematic of vortex based hydrodynamic cavitation reactor.

Vortex-based cavitation devices (VDs) overcome limitations of traditional orifice and venturi designs by eliminating flow constrictions, enabling cavitation away from walls and making them ideal for slurries [20] and delivering superior efficiency at equivalent power inputs [15], [17], [21]. A schematic of the VD is shown in Fig. 1, highlighting its constriction-free flow path [22]. Despite these advantages, scaling VDs remains challenging due to limited data on performance across scales of VD (d_t_ = 3 – 48 mm) and conditions (e.g., inlet pressure). While emerging applications like emulsification [23], [24] and crystallization [25], [26] demonstrate cavitation-induced intensification, their results are difficult to generalize across scales, particularly in water treatment, where quantifying OH• generation is of great importance. Chemical dosimetry, especially coumarin dosimetry allows quantification of OH• generation and thereby offers an indirect measure of cavitational activity [27], [28], [29], [30]. Most of the previously published research of cavitational activity generated by VD have been gathered based on degradation of pollutants (e.g., dyes and pharmaceuticals and other emerging pollutants). However, it does not provide unambiguous quantitative data on OH• generation. This work addresses this gap by analyzing published coumarin dosimetry datasets from De-Nasri *et al*. [15] and Sarvothaman *et al*. [31], enabling scale-dependent modeling of OH• generation in VDs. The study of De-Nasri *et al*. [15] reported data on an semi-industrial-scale HC reactor with one of the reactors having a nominal capacity of 200 Liters per minute (12 m^3^/hr). The definition of industrial scale reactor generally varies with the application. For example, typical industrial flow rates for anaerobic digester pre-treatment applications are of the order of 50 m^3^/h [32], while for applications such as distributed water treatment, flow rates are typically lower (∼10 m^3^/h). In this work, we have considered HC devices with nominal flow rates of ∼10 m^3^/h. The data and validated models presented here provide a sound basis for scaling up HC devices to real-world systems.

Given the complexity of VD performance, traditional models struggle to capture non-linear relationships across scales and operating conditions. Machine learning (ML) offers an attractive approach to address this challenge by uncovering hidden patterns and optimizing scale-up strategies [33], [34]. In recent years, ML has exhibited its strength and potential in chemical engineering, including prediction of rection outcomes and providing guidance for catalyst development [35], [36], [37]. This study applies a suite of supervised ML models to analyze the selected dataset spanning throat diameters (d_t_ = 6 – 38 mm: [nominal capacities translating to 5 to 200 L per minute (LPM)]), inlet pressures (100 – 400 kPa), and other operating conditions. The dataset leverages coumarin dosimetry to quantify cavitational activity via OH• generation performance, ensuring chemical specificity. Other reaction conditions, including pH, acid type, and temperature, are captured, and analyzed by ML models to identify their impact on performance. Previous work, such as Ranade *et al*. [38] have utilized artificial neural networks to explore performance of two different applications of HC – biomass pretreatment and water treatment, yet its investigation of scale-up considerations relies on a limited dataset, a single model, and indirect metrics based on pollutant disappearance. In contrast, this study leverages a broader ML ensemble on a chemically direct, multi-scale dataset to elucidate scale effects on radical yield and energy efficiency. The resulting predictive framework delivers actionable design guidelines for VDs on multiple scales, empowering researchers, and engineers to optimize device selection and operating conditions across water treatment—and broader applications in food, energy, and process intensification.

## 2 Experiments and calculation procedure

### 2.1 Standardization for Assessing HC reactor performance

To evaluate the performance of VD in terms of cavitational activity, coumarin is an appropriate probe molecule, and has been used as a chemical dosimeter [39], [40], [41], [42]. The formation of 7-hydroxycoumarin (7OHC), a hydroxylation product of coumarin, serves as an indicator of OH• generation performance. The production of 7OHC was measured in relation to the number of passes through hydrodynamic cavitation reactors with three different throat diameters: 6, 12 and 38-mm (nominal capacities: 5, 20 and 200 LPM respectively) [15], [16], [31]. Other experimental conditions, such as the type of acid, pH, initial concentrations considered may influence the overall performance. A summary of the two source datasets is provided in Table 1.

**Table 1:** Existing studies using coumarin dosimetry investigating VD.

Model system	Throat diameter (d_t_-mm)	Inlet pressure studied(kPa)	Acid used	pH	Operation	Reference
Wastewater treatment (Coumarin in water)	6, 12 and 38	200, 200 and 280 respectively	H_2_SO_4_	Acidic(3)	Isothermal (18, 18, and 26 °C)	De-Nasri *et al*. [15]
Wastewater treatment (Coumarin in water)	6 and 12	100 – 400	HCl	Acidic(3)	Non-isothermal (18 to 38 °C)	Sarvothaman *et al*. [31]

The target variable used is the concentration of 7OHC (µM), which describes the overall behaviour of hydrodynamic cavitation reactors, and such an approach has been used before [43], [44], [45]. It is important to note that chemical dosimetry methods including coumarin, salicylic acid, terephthalic acid, iodide, and Fricke systems—differ in selectivity, sensitivity, and operational constraints (e.g., pH and detection techniques). Moreover, the measured signal reflects not only hydroxyl radical generation but also probe-specific reaction pathways, product yields, and analytical methodologies. In the present work, coumarin dosimetry has been employed within a consistent and internally comparable framework. Where literature data from other systems are incorporated, appropriate normalization procedures and careful interpretation have been applied. The aim is not to assert absolute equivalence of OH• yields across different systems, but rather to identify trends and relative variations with scale.

Previous studies of HC reactors clearly show time (t) alone is not a good indicator to base performance analysis, the number of passes n (−) was introduced [46]. This overall performance can be interpreted with respect to process time, number of passes (Equation (1)) as:

(1) $$n\;\left(-\right)=\;\frac{Q\times t}{V}$$

Other approaches such as the ‘characteristic number of passes’ (Equation (2)) exist, which attempt to quantify the performance as the time spent in the cavitation zone, however, the data of this approach is limited to only d_t_ = 6 and 12-mm [31] and needs to be expanded to simulate VD performance for larger scales.

(2) $$n^{\prime }=\;\frac{v_{t}\times t}{d_{t}}n^{*}=\;\frac{n^{\prime }}{60000}$$

### 2.2 Dataset Preparation

Two datasets were from studies [15], [31]. While the dataset is modest in size, 167 datapoints in total, it encompasses a wide variety of experimental conditions, and it is suggested that relatively small datasets can achieve comparable results [47]. The experimental conditions are summarized in Table 1. They were merged to develop the ML model, with performance trends for d_t_ = 6 and 12 mm plotted in SI Fig. 1.

Sarvothaman *et al*. [31] used hydrochloric acid (HCl) at pH = 3 under non-isothermal conditions, varying inlet pressure (P_1_ = 100 – 400 kPa) for d_t_ = 6 and 12 mm, to obtain their OH• generation data. This dataset reported duplicates for the d_t_ = 12 mm reactor with an uncertainty in data points within 5 %, and no duplicates for the d_t_ = 6 mm reactor. De-Nasri *et al*. [15] used sulfuric acid (H_2_SO_4_) at pH 3 under isothermal conditions, covering d_t_ = 6 – 38 mm at P_1_ = 200–280 kPa, to obtain their OH• generation data. All the data in this study reported duplicates with an uncertainty in data points within 5 %. Both the studies [15] and [31] used distilled water as the liquid medium for carrying out coumarin dosimetry. Volume (V), pH and d_t_ are direct experimental conditions and are used as features for the following research. P_1_ and P_2_ were important experimental conditions. However, compared with the specific values of P_1_ and P_2_, pressure drop (ΔP = P_1_ – P_2_) is the more critical determinant. Accordingly, P_2_ and ΔP were extracted as features. Except for these, the differences in experimental conditions need to be extracted as features, including the type of acid and use of a cooling jacket. The effectiveness of hydrochloric and sulfuric acid in generating hydroxyl radicals differs. The study by Gągol *et al*. [48] demonstrates that inorganic acids affect degradation efficiency beyond simple pH effects, with sulfate-containing systems exhibiting markedly different behaviour compared to chloride-based systems. This divergence is attributed to the presence of multiple anionic species and their involvement in radical-mediated pathways, rather than to pH alone. In our formulation, the “number of anions” is employed as a simplified descriptor to differentiate such systems under identical pH conditions, where conventional parameters (e.g., pH or ionic strength alone) fail to fully capture these distinctions. For reactions conducted under isothermal conditions, temperature changes were negligible. In contrast, reactions performed without temperature control exhibited varying degrees of temperature rise. Therefore, temperature variation (ΔT) was also extracted as a feature. ‘n (−)’ is extracted as a feature to capture the reaction time and was calculated for these two datasets. The target variable is [7OHC], which is a surrogate indicator of OH• generation.

From the previous studies of VD, the throat diameter and corresponding nominal flowrates have been extracted [16]. A correlation between the flow capacity (m^3^/hr) and throat diameter (mm) is proposed in Eq. (3) based on the values of throat diameter and flowrate of the d_t_ = 6, 12 and 38-mm (this relationship, see SI Fig. 2).

(3) $$Q\left(m^{3}/hr\right)=\frac{{d_{t}}^{2}}{145}\left(d_{t}\;in\;mm\right)$$

### 2.3 Machine learning modelling

The full test dataset, comprising 15 % of the data, was manually separated at the outset and kept entirely independent during training. The remaining data were randomly split, with 85 % used for training and 15 % for validation. Seven commonly supervised machine learning algorithms were utilized with the datasets: Linear Regression (LR), Artificial Neural Network (ANN), K-Nearest Neighbours (KNN), Decision Tree (DT), Random Forest (RF), Gradient Boosting (GB), and eXtreme Gradient Boost (XGB). All of these algorithms are supervised learning methods, which rely on labeled data during training to generate accurate predictions. To evaluate the performance of the models developed, the coefficient of determination (R^2^) and root mean square error (RMSE) were employed as metrics as shown in Equations (4), (5):

(4) $$R^{2}=1-\frac{\sum_{m=1}^{M}{\left(\hat{y}-y\right)}^{2}}{{\left(\hat{y}-\overset{¯}{y}\right)}^{2}}$$

(5) $$\mathrm{RMSE}=\sqrt{\frac{\sum_{m=1}^{M}{\left(\hat{y}-y\right)}^{2}}{m}}$$

where y, $\overset{¯}{y}$ and $\hat{y}$ denote the actual, averaged and predicted values of the target feature, respectively, m represents the data point for an instance, and M is the total number of data points. A higher R^2^ and a lower RMSE indicate better model performance.

## 3 Results and discussion

### 3.1 Rationale and description for considering two diverse datasets

**Fig. 2:**
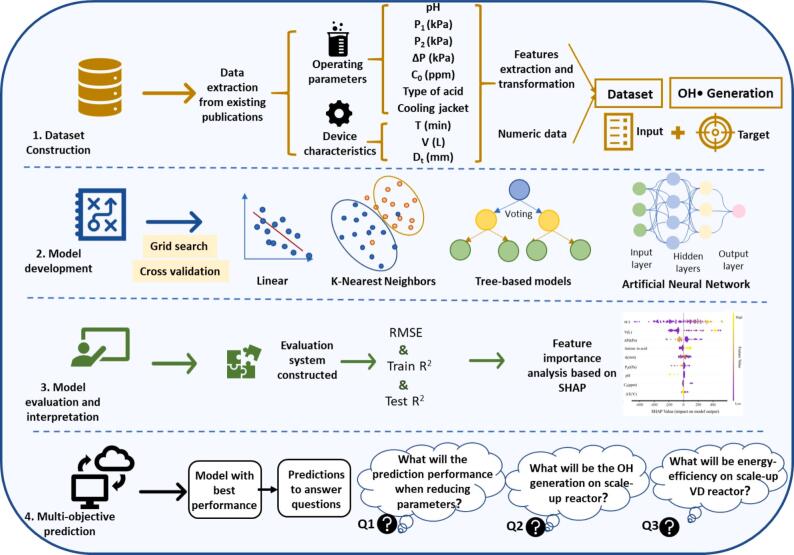
Schematic of the workflow of this study.

The two key studies in consideration for the development of the ML model are tabulated in Table 1, and the trends of the d_t_ = 6 and 12-mm reactors are plotted in SI Fig. 1. The first study offers an insight into a range of relevant inlet pressure (P_1_ = 100 to 400 kPa) for two VDs having d_t_ = 6 and 12-mm [see SI Fig. 1 (a) and (b)]. Whereas the second study investigated a larger range of d_t_, ranging from 6 to 38 mm. Since the study by Sarvothaman *et al*. [31] investigates multiple operating pressure, and the study by De-Nasri *et al*. [15] studied a larger range of d_t_, these were merged to derive predictions of interpolation on the influence of scale after the confirmation of the best machine learning model. The workflow of this study is summarized in Fig. 2.

### 3.2 Model training and evaluation

**Fig. 3:**
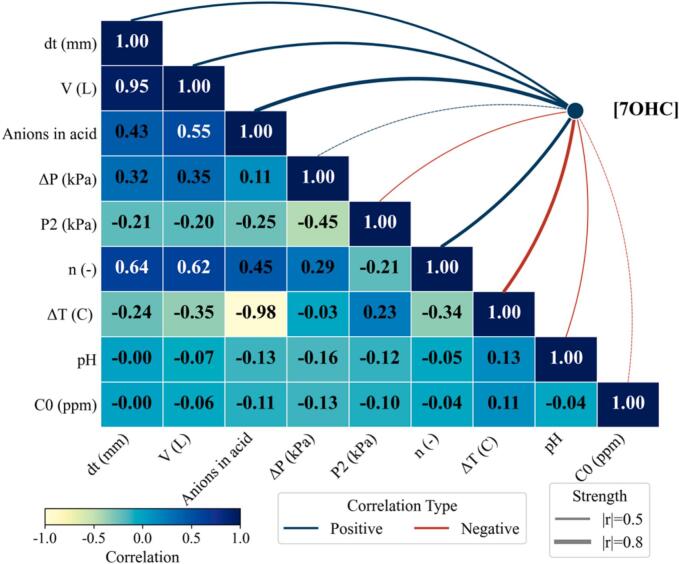
Pearson correlation matrix between all features.

A preliminary exploration of the correlation between all features was carried out and the Pearson correlation coefficient (PCC) are presented in Fig. 3. The PCC, measures the strength and direction of the linear relationship between two continuous variables: values close to 0 indicate little or no linear association, whereas values near 1 or −1 reflect strong positive or negative linear relevance, respectively. The corresponding color–coded matrix is also presented, with dark blue representing a value of 1 and yellow representing − 1. Fig. 3 shows that the PCC of anions of the acid, n (−) and temperature rise on the target were relatively higher than the others, with the lines were stronger than the others, implying a strong correlation. The PCC between the d_t_ and the V was very high (0.95), primarily due to the reason that when employed on a larger volume, higher d_t_ is expected. The PCC between the anions in acid and the temperature rise was very high as well (−0.98), primarily due to the complete distinction in experimental conditions for these two factors between two datasets. However, this does not necessarily indicate a strong inherent correlation between these two factors. The correlation between other variables was small and requires further model calculation for specific correlation analysis.

**Fig. 4:**
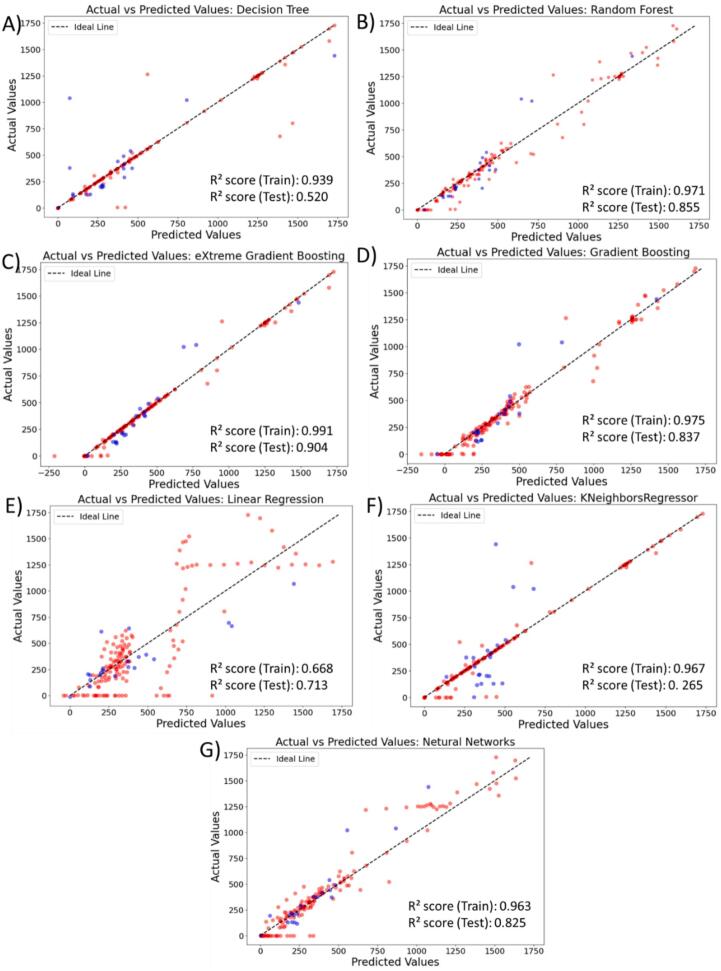
Predicted scatter plot with seven ML models [*x*-axis represented the experimental values, *y*-axis represented the predicted result, Red symbols = training dataset, Blue symbols = test dataset].

The dataset described previously was utilized to train seven machine learning models. Model performance was assessed by predicting OH• generation, with results visualized in a scatter plot comparing actual and predicted values (Fig. 4). All the R^2^ values were calculated on a training set and a test set and the numbers are indicated on Fig. 4. It shows that higher R^2^ values were observed on the training set compared to the test set on all the models except for linear regression, indicating some degree of overfitting despite the use of cross-validation and grid search to mitigate this issue. The R^2^ values on LR are relatively low, that are 0.668 and 0.713, respectively. The R^2^ values of DT, RF, GB, XGB, KNN and ANN on the training set were over 0.9. However, their performance on the test set varied significantly. The DT and KNN models exhibited notably lower R^2^ values of 0.520 and 0.265, respectively, indicating poorer generalization. Conversely, the RF, GB, ANN, and XGB models maintained relatively high R^2^ values on the test set, at 0.855, 0.837, 0.825, and 0.904, respectively. Among all models, XGB demonstrated superior predictive performance, achieving the highest R^2^ values on both the training and test sets.

**Fig. 5:**
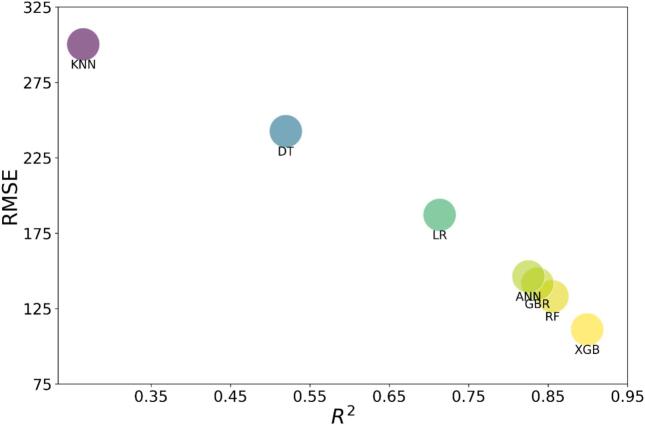
Comparison of Model Performance: R^2^ and RMSE.

In addition to R^2^, the RMSE was used to evaluate machine learning model performance by measuring the average error between predicted and actual values. Lower RMSE indicates better accuracy and smaller deviations from observed data. Comparing RMSE across models helps assess their effectiveness in capturing dataset patterns. The R^2^ and RMSE values on the training set for the seven models are summarized in Fig. 5. It shows that among the seven machine learning algorithms, three tree-based algorithms RF, GB, and XGB had R^2^ test > 0. 8 and relatively lower RMSE, indicating their good performance on the training set. Compared with these models, DT, LR and KNN have a lower R^2^ and higher RMSE even after optimization, indicating their unsuitability for further predictive tasks. XGB was selected as the representative tree-based model. The performance of the ANN was suboptimal among these models, and thus it was selected as the representative non–tree-based counterpart. Both models are excellent models at ML, while XGB is reported to have a better performance at prediction when a relatively small dataset was used than ANN [49]. Consequently, OH• generation in cavitation devices were predicted and analysed by evaluating and comparing these two models.

**Fig. 6:**
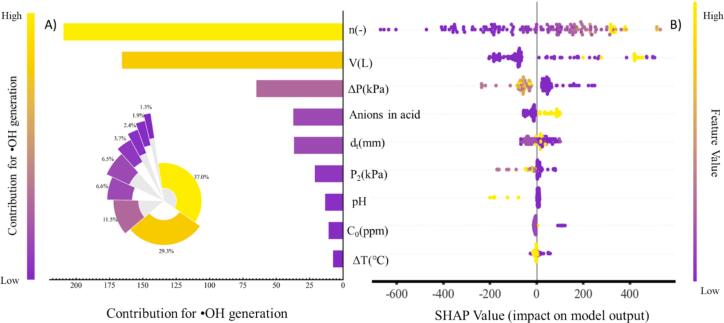
Feature importance analysis for XGB models, based on SHAP analysis – A): Features contribution on OH• generation. B): SHAP analysis based on model output.

**Fig. 7:**
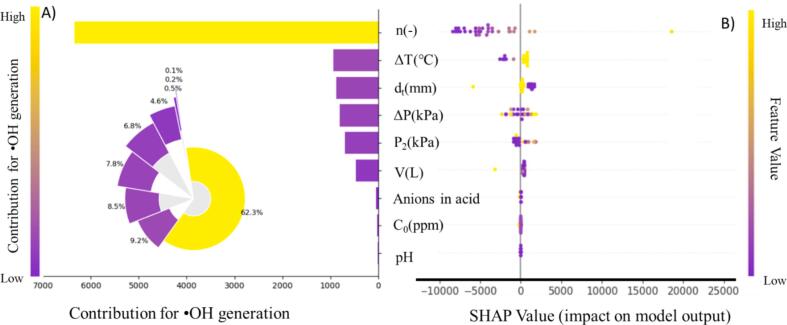
Feature importance analysis for ANN models, based on SHAP analysis – A): Features contribution on OH• generation. B): SHAP analysis based on model output.

Shapley values (SHAP) method was employed for interpretation of the XGB and ANN models. SHAP analysis, serves as a robust and model-agnostic methodology, enables the interpretation of machine learning models by quantifying the contribution of each feature to individual predictions. Grounded in cooperative game theory, SHAP values allocate a unique importance score to each feature, reflecting its average marginal contribution across all possible feature combinations, thereby ensuring a fair and consistent distribution of predictive influence. This approach delivers both local interpretabilities, clarifying the rationale behind individual predictions, and global insights, elucidating overarching trends in feature importance across the dataset. The SHAP analysis of the XGB model and ANN model are shown in Fig. 6 and Fig. 7, respectively. As shown in both figures, both the XGB and ANN model identified n (−) as a critical feature for predicting 7OHC concentration, which is consistent with expectations [48].

The XGB model’s importance analysis ranked V, ΔP, anions in acid and P_2_ as the next most critical predictors. And the 5 features count for over 90 % in important analysis. ANN model’s importance analysis ranked ΔT, d_t_, ΔP, V, and P_2_ as the next most critical predictors. The importance analysis on ANN model shows that n (−) accounts for 62.3 %, significantly outweighing other features. This prominence may stem from the scaling process applied to features in ANN models. Scaling can amplify the importance of features with smaller variance by emphasizing their relative changes in the transformed space. This effect will become evident in SHAP value analyses, where scaled features may exhibit inflated contributions due to the model's sensitivity to their transformed values. To evaluate this, a neural network model without feature scaling was also trained and the SHAP analysis indicates that the feature importance accounts for 46.4 % (results not shown). However, the R^2^ (test) on ANN without feature scaling is significantly lower (0.638 compared to 0.825 with feature scaling), highlighting the critical role of feature scaling in improving ANN performance.

**Fig. 8:**
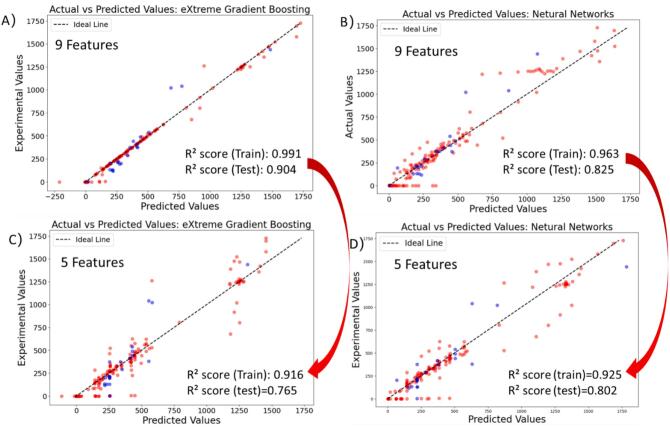
The comparison of the predicted scatter plot with XGB and ANN models with 9 features and 5 features, respectively. A): XGB model with 9 features; B): ANN model with 9 features; C): XGB model with 5 features; D): ANN model with 5 features. (Red symbols = training dataset, Blue symbols = test dataset).

The analysis of feature importance in XGB model reveals that the top five features collectively account for over 90 % of the total importance. And these five features also account for over 90 % of ANN models. Using a reduced set of high-importance features can lower model complexity and computational costs and therefore, it is worthwhile evaluating the model's performance when utilizing only these top five features, with the hyperparameter re-tuned. The results were summarized and shown in Fig. 8.

The R^2^ (Test) for the XGB model dropped significantly from 0.904 to 0.765, while the ANN model's R^2^(Test) decreased from 0.825 to 0.802, indicating a more substantial performance decline in XGB compared to the ANN model. XGB is a gradient boosting model based on decision trees, which rely on the combined effects of features to capture complex nonlinear relationships. Removing some features (even those with lower importance) may disrupt these combined effects, making the model fail to fully utilize the information in the data, and thus significantly reduce performance. In contrast, ANN can extract complex patterns from fewer features through multilayer nonlinear transformations. Even when using only the top 5 features, neural networks may still capture key patterns through their deep structures and weight adjustments, resulting in less noticeable performance degradation. Due to the substantial performance drop in XGB model, all original features will be retained for subsequent predictions to ensure optimal model performance.

### 3.3 Model-Based interpolations

**Fig. 9:**
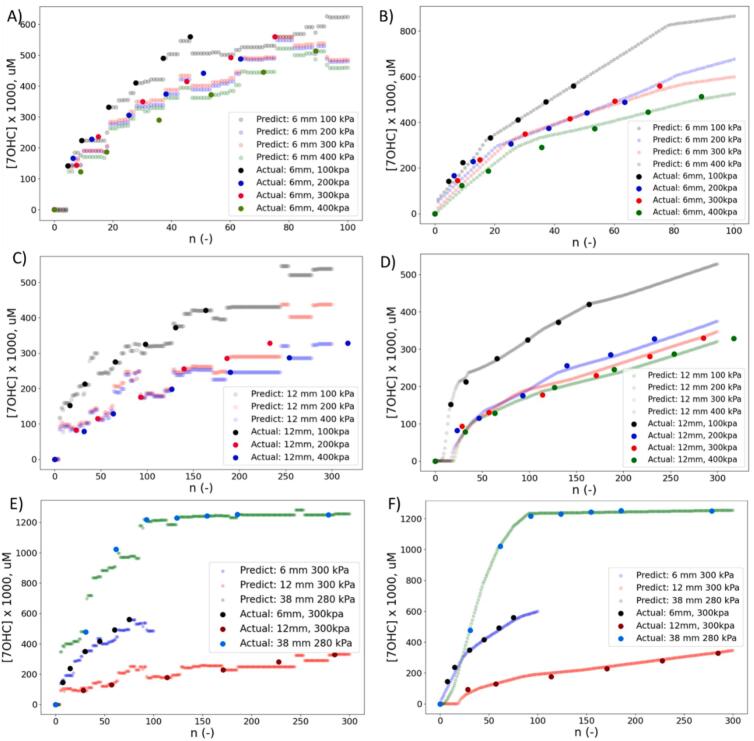
Predicted results using XGB and ANN models compared with experimental data. A) XGB predictions for a 6 mm reactor at varying ΔP. B) ANN predictions for a 6 mm reactor at varying ΔP. C) XGB predictions for a 12 mm reactor at varying ΔP. D) ANN predictions for a 12 mm reactor at varying ΔP. E) XGB predictions at around 300 ΔP across different throat diameters. F) ANN predictions at around 300 ΔP across different throat diameters.

After the confirmation of the excellent performance of the developed machine learning models in representing experimental data, their interpolation capabilities were examined. Simulations were conducted to predict output variables for unknown input conditions within the range of the experimentally investigated parameters with XGB and ANN models. The available experimental data were acquired through scheduled sampling, so the n (−) values were not continuous. As an initial step to assess model reliability, n (−) was augmented via interpolation, to achieve full coverage. Specifically, for d_t_ = 6 mm, n (−) range from 1 to 100, and d_t_ = for 12 mm, n (−) ranged from 1 to 300. The results are summarized in Fig. 9.

The experimental data and predicted data are both plotted on this figure, and it shows that the actual experimental data closely align with the predicted trend line. Predictions from the XGB model display a step-like behavior, owing to its tree-based architecture. Trees partition the feature space into regions (leaves). Every sample that falls in the same leaf gets the same prediction. Gradient boosting combines multiple decision trees, with each tree giving an output of a constant value per leaf. The sum is therefore piecewise constant. When the predictions move along with a feature [e.g., n (−)], the prediction only changes when you cross a split threshold. And that makes the predictions look like steps. Typical ANNs use continuous activations [tan (h) used in this study]. The mapping from inputs to output is continuous, so small input changes produce small output changes. That makes the prediction line look likes smooth curves. It must be noted that smoothness is an aesthetic/property of the hypothesis class, not accuracy.

**Fig. 10:**
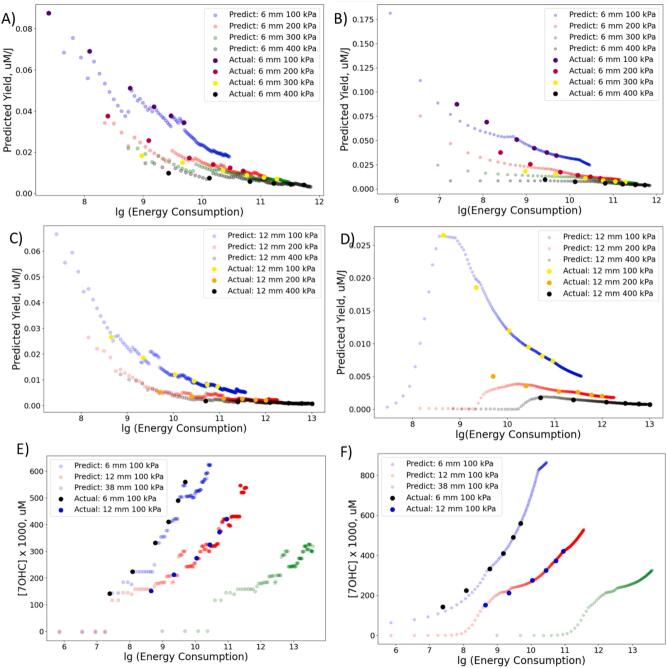
Analysis of product yield and OH• concentration versus energy consumption for different reactor sizes, pressure drops, and throat diameters. A) XGB predictions for product yield in 6-mm reactors with varying ΔP; B) ANN predictions for product yield in 6-mm reactors with varying ΔP; C) XGB predictions for product yield in 12-mm reactors with varying ΔP; D) ANN predictions for product yield in 12-mm reactors with varying ΔP; E) XGB predictions for OH• concentration in reactors with 100 ΔP and varying throat diameters; F) ANN predictions for OH• concentration in reactors with 100 ΔP and varying throat diameters.

As the next step, it is important to evaluate the product yield with respect to energy. Analysing energy consumption versus hydroxyl radical generation is a critical aspect of evaluating and optimizing advanced oxidation processes, which are widely used in water and wastewater treatment to degrade recalcitrant organic pollutants, pharmaceuticals, dyes, and microbes. The comparison helps quantify how efficiently energy inputs translate into OH• production and, ultimately, pollutant removal efficiency. The yield with respect to energy consumption was simulated for d_t_ = 6 and 12-mm, for P_1_ = 100 to 400 kPa, and they were calculated using Eqs. (6) and (7). Additionally, the yield for d_t_ = 6 and 12-mm at P_1_ = 100 kPa is compared to the d_t_ = 38-mm (Fig. 10A – D). The plots with the simulated trends are validated against experimental data where possible and showed a good agreement. What can be seen is that the VD regardless of scale exhibit an earlier optimal performance contrary to that of conventional cavitation reactors, and that the efficiency is higher for a smaller scale of VD. Moreover, from Fig. 10 (E) and From Fig. 10 (F), it is also shown that, with same energy consumption, higher concentration of OH• is observed in smaller throat diameter. From an energetic standpoint, a smaller throat diameter is typically more favourable due to its potential to optimize energy efficiency. However, a smaller diameter is often less suitable for processing large volumes of water, necessitating a careful balance between energy optimization and throughput capacity for device-design.

(6) Energy consumption =ΔP × n (-) × V

(7) $$\mathrm{Yield}=\frac{\left[7OHC\right]}{\text{E}\text{nergy consumption}}$$

After confirming that the XGB and ANN models accurately described the available experimental data and successfully interpolated results within the considered parameter range, it was worthwhile to evaluate their performance in extrapolation. Investigating the impact of scale-up on hydrodynamic cavitation is crucial for the design of effective devices. The range of diameter of the vortex-diode of the available data was only from 6 mm to 38 mm. And therefore, these two models were used to simulate the radical yield by changing throat diameter from 3 mm to 100 mm. By plotting this relationship across many throat diameters, the model eliminates the necessity for physical testing of each configuration, which is particularly valuable given the non-linear and complex nature of cavitation dynamics [50]. And the results are summarized and plotted in Fig. 10.

The available data of XGB and ANN pertain to the formation of radicals which is from coumarin oxidation (simulated trend, d_t_ = 3 to 72-mm), whereas the data on scale-up is that of dichloroaniline degradation (experimental trend, d_t_ = 3 to 38-mm). Moreover, the data of dichloroaniline degradation was gathered at a pH of 7, and coumarin oxidation was carried out at pH of 3. In order to normalize this, there are two factors to be considered - the first factor being the available experimental datasets for DCA and coumarin correspond to different pH conditions, and pH is known to significantly influence radical generation and reaction kinetics in cavitation-based advanced oxidation processes. The second factor being, coumarin hydroxylation and DCA degradation have different intrinsic reaction rate constants with hydroxyl radicals, which necessitates scaling when comparing absolute rates. Based on kinetics of DCA degradation using oxidative mechanisms similar to that of hydrodynamic cavitation, it can be inferred that a ∼ 2.6 time faster degradation can be expected at pH 3 when compared to pH 7 for DCA [51], [52]. The use of a single scaling factor here is a simplification and may not fully capture differences in reaction pathways or kinetics between probe molecules. However, the current approach suffices to provide a semi-quantitative validation of trends, rather than exact quantitative prediction. Future work will focus on generating consistent datasets for a single probe molecule across controlled pH conditions to enable more rigorous validation.

**Fig. 11:**
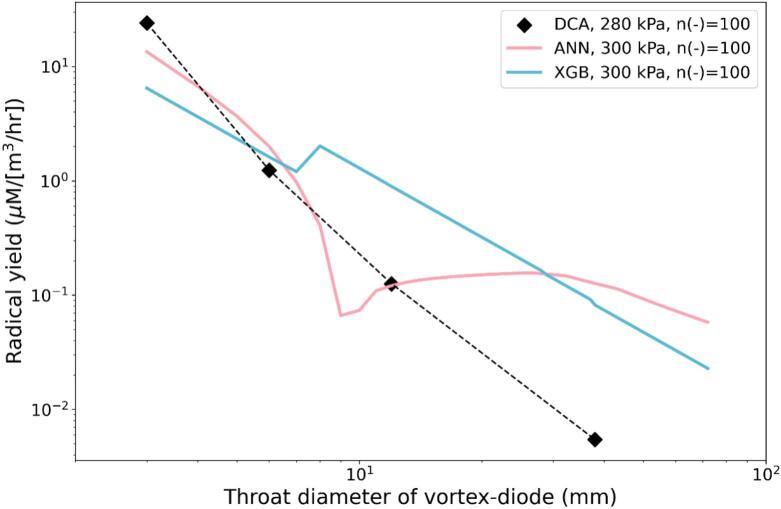
The relationship between the throat diameter and the radical yield for ANN, XGB models and experimental data, when n (−) = 100, DCA degradation data from Ranade et al. [16].

Upon applying this normalization technique, the experimental data was plotted as a trend on the available ANN and XGB curves. The XGB and ANN models exhibit consistent trends in their predictions, with increasing throat diameter, the radical yield decreased. On a closer examination, a good qualitative agreement with the experimental trend is obtained for the XGB simulated curve beyond 10-mm, however, the ANN trend shows a saddle around d_t_ value of 10-mm and a plateau in performance beyond it with poor qualitative agreement with experimental data. A significant deviation between the two models, however, is observed in the vicinity of throat diameter around 10-mm (Fig. 11). This discrepancy may arise from differences in feature representation, model inductive biases, and limited coverage of the chemical space in the training data. Such divergence highlights a known limitation of purely data-driven ML approaches: predictions can vary between models. One approach to overcome this limitation is to generate predictions from multiple models and aggregate or compare their outputs, thereby achieving more robust and reliable predictions.

The XGB trend is more representative of experimental data. The predictions generated by XGB model, elucidating the relationship between throat diameter and hydroxyl radical yield in a hydrodynamic cavitation-based advanced oxidation process system are informative. It should be noted that a direct, quantitative relationship between scale and OH• generation is not yet fully established in the literature. Recently Khare and Ranade [53] have studied influence of scale of vortex-based cavitation devices on flow characteristics of cavitation zone. Results of this work together with the results of Khare and Ranade [53] provide a mechanistic framework to interpret the observed trend of reduction of effective OH radical yield with scale. Simulations and analysis show that, under geometrically similar scale-up:

(i) Cavitation extent (dimensionless vapor volume per unit input energy) decreases significantly with scale, indicating fewer effective cavitation events per unit energy input.
(ii) Specific energy dissipation rate decreases with increasing device size, even at comparable operating conditions.
(iii) Cavitation-zone characteristics—such as mean pressure, amplitude of pressure fluctuations, and turbulence frequency—change with scale, affecting both the intensity and dynamics of cavity collapse.

Since OH• generation is strongly linked to the intensity and frequency of cavity collapse, these changes collectively lead to a reduction in effective OH• production (Pandit *et al*. [54]). In particular, lower energy dissipation rates and altered pressure fluctuation characteristics imply weaker or less frequent high-energy collapse events, which are responsible for radical formation. In addition, although the total vapor volume may scale with device size, the radical yield per unit throughput (or per unit energy input) decreases due to reduced cavitation effectiveness, consistent with the observed decline in pollutant degradation performance. The precise coupling between cavitation hydrodynamics and radical generation, especially the role of micro-scale collapse dynamics and chemical kinetics remains an open research question.

Nevertheless, the presented trends in this study effectively address practical constraints associated with experimental reactor testing, enhance energy efficiency, inform cost-effective reactor design, and contribute to the development of sustainable water treatment solutions. By producing an extensive dataset, the model serves as a robust tool for investigating the intricate interplay between throat diameter and radical production. The digitized data (radical yield at throat diameter from 3 mm to 72 mm for ANN, XGB models) is summarized in SI Table 1. This is important in wastewater treatment applications, where energy costs and radical generation efficiency are pivotal determinants of practical feasibility and scalability.

## 4 Conclusions

Quantitative assessment of VD based hydrodynamic cavitation reactors across different scales and operating conditions is critical for optimizing reactor design and performance for various industrial applications. This study introduces a novel approach by employing ML models, specifically XGB and ANN, addressing a significant data gap in systematic data for reactors with nominal capacities between 20 and 100 LPM using coumarin dosimetry, to obtain OH) generation. By analysing datasets from two studies – the ML models revealed critical insights into device behaviour. Number of passes [n (−)] was used to compare these two datasets directly and overcome limitations of the original metric, enabling consistent performance comparisons across reactors with dissimilar volumes. SHAP analysis was employed to interpret and visualize the models. The results showed that both XGB and ANN models accurately simulate the data. Upon closer examination, the XGB was seen to have a better agreement with experimental data and trends of that prediction will be useful for HC device sizing to various applications. The innovative use of ML elucidated these scale-dependent trends. These advancements, driven by ML-driven interpolation and the extrapolation, provide a robust framework for future scale-up studies of vortex-diode HC devices and will facilitate implementation of scaled up devices in industrial applications with suitable validation, primarily in applications such as water treatment and emerging applications such as biomass or food processing.

## CRediT authorship contribution statement

**Xinzhu Pang:** Writing – original draft, Investigation, Formal analysis, Conceptualization. **Varaha P. Sarvothaman:** Writing – review & editing, Methodology, Investigation, Formal analysis. **Shekhar R. Kulkarni:** Formal analysis. **William L. Roberts:** Funding acquisition. **Vivek V. Ranade:** Writing – review & editing, Supervision, Methodology.

## Declaration of competing interest

The authors declare that they have no known competing financial interests or personal relationships that could have appeared to influence the work reported in this paper.
