# Supplementary material for: Scale-Dependent hydroxyl radical generation and energy efficiency in vortex diode hydrodynamic cavitation: machine learning insights toward industrial-scale applications in water treatment
Source: Ultrason Sonochem. 2026 Jun 22;131:107932. doi: 10.1016/j.ultsonch.2026.107932 (PMC13355199; doi:10.1016/j.ultsonch.2026.107932)
Supplement: Supplementary Data 1 [file mmc1.pdf]

Supplementary Information

Scale-Dependent Hydroxyl Radical Generation and Energy Efficiency in Vortex Diode Hydrodynamic Cavitation: Machine Learning Insights Toward Industrial-Scale Applications in Water Treatment

Xinzhu Pang[1,*], Varaha P. Sarvothaman[2,*], Shekhar R Kulkarni[2], William L Roberts[2], Vivek V Ranade[3,*]
[1]School of Forensic Medicine, China Medical University, No 77 Puhe Road, Shenyang North New Area, Shenyang, 110122, Liaoning, PR China.
[2]Clean Energy Research Platform (CERP), Physical Sciences and Engineering Division, King Abdullah University of Science and Technology (KAUST), Thuwal 23955-6900, Saudi Arabia.
[3]Multiphase Reactors and Intensification Group, Bernal Institute, University of Limerick, Limerick V94T9PX, Ireland.
*Corresponding authors: xzpang@cmu.edu.cn, varahaprasad.sarvothaman@kaust.edu.sa and vivek.ranade@ul.ie.

(a)

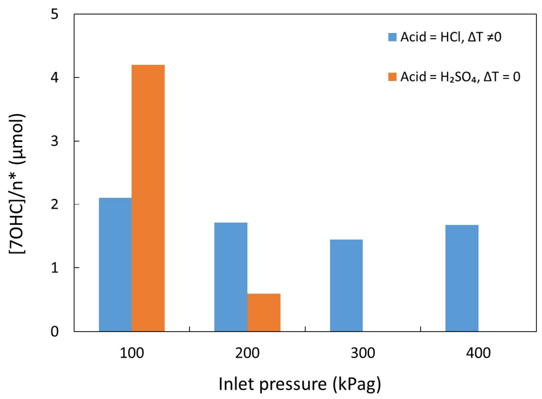

(b)

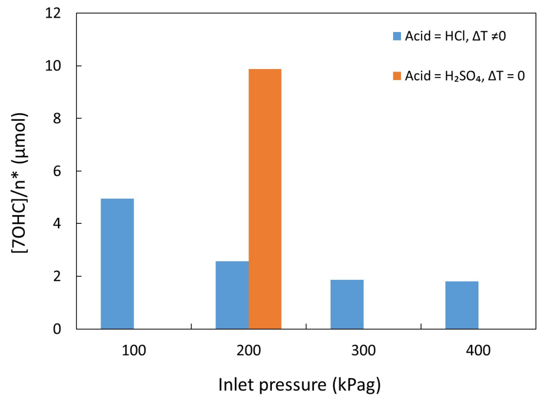

SI Figure 1: Influence of inlet pressure on 7-hydroxycoumarin formation rate for different throat diameters (d_t) (a) 6-mm and (b) 12-mm.

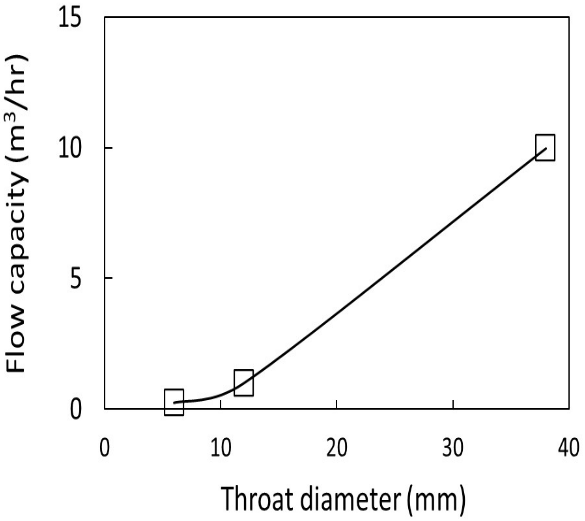

SI Figure 2: Capacity evaluation plot – relationship between throat diameter and flow capacity.

SI Table 1: Relationship between throat diameter and radical yield for ANN, XGB models and experimental data, for n (-) =100.

d_t (mm)	Radical yield: NN	Radical yield: XGB
3	13.47	6.47
4	6.72	3.64
5	3.67	2.33
6	2.00	1.62
7	0.98	1.20
8	0.41	2.02
9	0.07	1.60
10	0.07	1.29
11	0.11	1.07
12	0.12	0.89
13	0.13	0.76
14	0.13	0.66
15	0.14	0.57
16	0.14	0.50
17	0.14	0.45
18	0.15	0.40
19	0.15	0.36
20	0.15	0.32
21	0.15	0.29
22	0.15	0.27
23	0.15	0.24
24	0.16	0.22

25	0.16	0.21
26	0.16	0.19
27	0.16	0.18
28	0.15	0.16
29	0.15	0.15
30	0.15	0.14
31	0.15	0.13
32	0.15	0.12
33	0.14	0.12
34	0.14	0.11
35	0.14	0.10
36	0.13	0.10
37	0.13	0.09
38	0.13	0.08
39	0.12	0.08
40	0.12	0.07
41	0.12	0.07
42	0.12	0.07
43	0.11	0.06
44	0.11	0.06
45	0.11	0.06
46	0.10	0.06
47	0.10	0.05
48	0.10	0.05
49	0.10	0.05
50	0.09	0.05
51	0.09	0.05
52	0.09	0.04
53	0.09	0.04
54	0.08	0.04
55	0.08	0.04
56	0.08	0.04
57	0.08	0.04
58	0.08	0.04
59	0.07	0.03
60	0.07	0.03
61	0.07	0.03
62	0.07	0.03
63	0.07	0.03
64	0.07	0.03
65	0.07	0.03
66	0.06	0.03
67	0.06	0.03

68	0.06	0.03
69	0.06	0.02
70	0.06	0.02
71	0.06	0.02
72	0.06	0.02

Information on the hyperparameter tunings was only done for ANN and XGB models. The details of the hyperparameter are provided below:

Best XGBoost Parameters: {'colsample_bytree': 0.8, 'learning_rate': 0.05, 'max_depth': 8, 'n_estimators': 500, 'reg_alpha': 0, 'reg_lambda': 1.0, 'subsample': 0.7}

Best ANN Parameters: {'mlp__activation': 'relu', 'mlp__alpha': 1.0, 'mlp__early_stopping': False, 'mlp__hidden_layer_sizes': (200, 100), 'mlp__learning_rate_init': 0.05, 'mlp__max_iter': 2000, 'mlp__solver': 'adam'}

References:

[1]. S.J. De-Nasri, V.P. Sarvothaman, S. Nagarajan, P. Manesiotis, P.K.J. Robertson, V.V. Ranade, Quantifying OH radical generation in hydrodynamic cavitation via coumarin dosimetry: influence of operating parameters and cavitation devices, Ultrasonics Sonochemistry (2022) 106207.

[2]. V.P. Sarvothaman, S.R. Kulkarni, J. Subburaj, S.L. Hariharan, V.K. Velisoju, P. Castaño, P. Guida, D.M. Prabhudharwadkar, W.L. Roberts, Evaluating performance of vortex-diode based hydrodynamic cavitation device scale and pressure drop using coumarin dosimetry, Chemical Engineering Journal 481 (2024) 148593.
